# Use of Ursodeoxycholic Acid and Cancer Risk for Patients With Primary Biliary Cholangitis

**DOI:** 10.1001/jamanetworkopen.2025.50907

**Published:** 2025-12-19

**Authors:** Lyra Su, Parth Jagrut Patel, Xiang Shu

**Affiliations:** 1 Department of Epidemiology and Biostatistics, Memorial Sloan Kettering Cancer Center, New York, New York; 2 Rowan-Virtua School of Osteopathic Medicine, Stratford, New Jersey

## Abstract

This cohort study uses electronic health record data to compare cancer incidence among adults with primary biliary cholangitis who were or were not treated with ursodeoxycholic acid (UDCA).

## Introduction

Ursodeoxycholic acid (UDCA) is standard first-line therapy for primary biliary cholangitis (PBC), delaying liver damage and improving survival. However, its association with cancer risk remains controversial.^1^ Previous studies were limited by sample size and mixed confounding. We conducted a cohort study using electronic health record (EHR) data comparing cancer incidence among adults with PBC treated with UDCA vs nonusers.

## Methods

We used the TriNetX platform^2^ to access deidentified medical records of 156 million patients in the Research Network, including over 48 000 patients with PBC from 109 health care organizations worldwide. The University of Maryland institutional review board exempted this study from review because it does not constitute human participants research. The TriNetX platform has been widely used for retrospective cohort analyses of medication use and cancer risk.^3,4^ This cohort study followed the STROBE reporting guideline.

The study included patients with PBC (aged 18-89 years) without prior cancer and with no death record within 1 year of diagnosis (2005-2025). These patients were divided into 2 cohorts: (1) UDCA cohort, including patients with first UDCA prescription within first 6 months of PBC diagnosis, and (2) non-UDCA cohort. Cohorts underwent propensity score matching (PSM; 1:1 using nearest neighbor greedy matching) for demographic characteristics; adverse socioeconomic determinants of health; preexisting medical conditions (including liver cirrhosis and viral hepatitis); family and/or personal history of cancer, colonic polyp, and other benign neoplasms; problems related to lifestyles; medication use; and surgical procedures of the digestive system (Table). Cox proportional hazards regression models with hazard ratios (HRs) and 95% CIs were used to compare hazard rates, and log-rank *P* values were presented. The outcomes of interest included first primary diagnosis of gastrointestinal (overall and separately by colorectal and liver cancer) and breast cancer that occurred at least 1 year from the index event (ie, first prescription of UDCA for UDCA cohort or PBC diagnosis for nonusers). We performed additional Cox proportional hazards regression models on pre-PSM data with extensive covariate adjustment (eMethods in Supplement 1). The dataset was frozen on July 17, 2025. Analysis was conducted within the TriNetX Analytics Platform using built-in functions (R, version 4.0.2). Statistical significance was set at 2-sided *P* < .05.

**Table.** Demographic Characteristics of the UDCA and Non-UDCA Cohorts Before and After PSM^a^

Characteristic	Before PSM, No. (%)		After PSM, No. (%)	
	UDCA (n = 7509)	Non-UDCA (n = 11 656)	UDCA (n = 6057)	Non-UDCA (n = 6057)
Age at index event, mean (SD), y	58.1 (13.3)	55.9 (14.9)	57.5 (13.4)	57.3 (14.5)
Sex				
Female	6352 (84.6)	8439 (72.4)	4949 (81.7)	4949 (81.7)
Male	969 (12.9)	2867 (24.6)	939 (15.5)	957 (15.8)
Unknown	188 (2.5)	361 (3.1)	170 (2.8)	145 (2.4)
Ethnicity				
Hispanic or Latino	818 (10.9)	1201 (10.3)	660 (10.9)	654 (10.8)
Not Hispanic or Latino	4768 (63.5)	6924 (59.4)	3737 (61.7)	3792 (62.6)
Unknown	1922 (25.6)	3532 (30.3)	1660 (27.4)	1611 (26.6)
Race				
American Indian or Alaska Native	53 (0.7)	70 (0.6)	36 (0.6)	30 (0.5)
Asian	338 (4.5)	804 (5.9)	303 (5.0)	291 (4.8)
Black or African American	428 (5.7)	921 (7.9)	400 (6.6)	382 (6.3)
Native Hawaiian or Other Pacific Islander	23 (0.3)	58 (0.5)	18 (0.3)	18 (0.3)
Other	405 (5.4)	478 (4.1)	297 (4.9)	297 (4.9)
White	5234 (69.7)	7705 (66.1)	4137 (68.3)	4173 (68.9)
Unknown	1029 (13.7)	1725 (14.8)	866 (14.3)	866 (14.3)

Abbreviations: PSM, propensity score matching; UDCA, ursodeoxycholic acid.

^a^ The status of variables was based on the presence of related clinical codes anytime to 1 day before the index event. Other variables that were not shown but propensity score matched between cohorts include adverse socioeconomic determinants of health (eg, housing and economic circumstance, upbringing, educational level, physical environment, social environment); family circumstance; family and personal history of cancer; family and/or personal history of colonic polyp and other benign neoplasms; family history of colorectal cancer; problems related to lifestyles (eg, exercise, diet, smoking, alcohol drinking); and preexisting medical conditions and procedures, including overweight and obesity, liver cirrhosis, viral hepatitis, Crohn disease, ulcerative colitis, cystic fibrosis, bariatric surgery, and colonoscopy. For breast cancer, the propensity score matching was further tailored to include mammography test and hormone replacement treatment.

## Results

After 1:1 PSM, 6057 patients with PBC were included in each cohort. Mean (SD) age at index was 57.5 (13.4) years for UDCA users (81.7% women and 15.5% men) and 57.3 (14.5) years for nonusers (81.7% women and 15.8% men; Table). Most UDCA users (4882 of 7435 [65.7%]; pre-PSM) had first prescription within 1 day (mean [SD], 17 [38] days to first prescription). Patients with UDCA treatment showed significantly lower risk of gastrointestinal cancer than nonusers (HR, 0.59; 95% CI, 0.43-0.80) (Figure, A). Similar associations were observed for liver cancer (HR, 0.54; 95% CI, 0.37-0.77) and breast cancer among females (HR, 0.36; 95% CI, 0.18-0.72). There was no association between use of UCDA and development of colorectal cancer (HR, 0.54; 95% CI, 0.25-1.20). Comparable results were obtained in Cox proportional hazards regression models using pre-PSM data for all 4 cancer types (Figure, B). We performed sensitivity analysis by defining the index event as the date of PBC diagnosis for both cohorts, yielding similar but less significant results.

**Figure.**
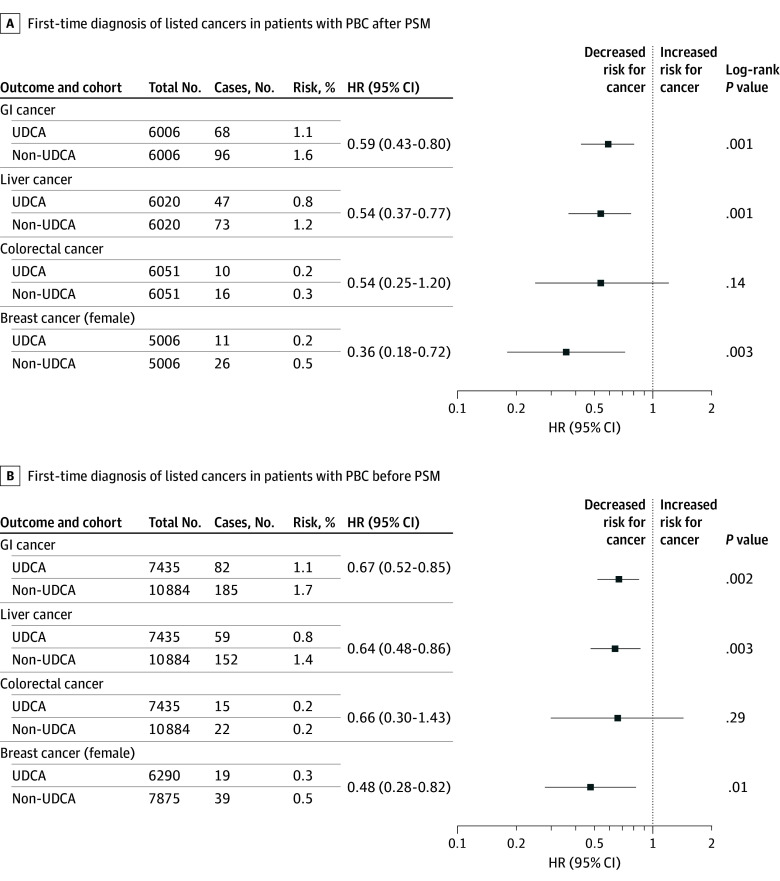
Associations Between Use of Ursodeoxycholic Acid (UDCA) and Cancer Risk Overall risk and hazard ratios (HRs) for UDCA vs non-UDCA of first-time diagnosis of listed cancers in patients with primary biliary cholangitis (PBC) after (A) and before (B) propensity score matching (PSM). Risk (%) refers to the fraction of patients who developed the outcome of interest in the time window (risk = patients with outcome/patients in cohort). Cases refers to the number of incident cancer events after PSM. For breast cancer, additional covariates were used for PSM, including history of breast cancer screening mammography and diagnostic mammography, oral contraceptive use, and use of hormone replacement therapy. *P* values in panel A are log-rank *P* values, while the *P* values in panel B are multivariable Cox proportional hazards regression *P* values. GI indicates gastrointestinal.

## Discussion

In this study, UDCA treatment was associated with significantly lower risks of gastrointestinal cancer, liver cancer, and breast cancer but not colorectal cancer in patients with PBC. Strengths of the study include large sample size, robust confounder control, minimized immortal time bias, and harmonized EHR data. Limitations include residual confounding, self-selection bias, lack of data on drug use duration and compliance, and potentially insufficient follow-up time to capture long-term cancer outcomes.^5^

Our findings suggest a potential chemopreventive effect of UDCA beyond its hepatic benefits, consistent with some prior reports.^1^ The underlying protective mechanism of UDCA remains to be elucidated but is presumably related to its anti-inflammatory, antiproliferative, and cytoprotective properties.^1^ Confirmation of our findings using prospective studies or randomized clinical trials is warranted, particularly for liver and breast cancer.

## Notes

Supplement 1.
eMethods.

Supplement 2.
Data Sharing Statement
