# Errors in Byline and Location of Affiliation

**DOI:** 10.1001/jamanetworkopen.2026.2060

**Published:** 2026-02-23

In the Research Letter titled “Use of Ursodeoxycholic Acid and Cancer Risk for Patients With Primary Biliary Cholangitis,”^1^ published December 19, 2025, there was an error in the degree listed for Parth Jagrut Patel and in the location of his affiliation. This article has been corrected.^1^
