# Supplementary material for: Use of Ursodeoxycholic Acid and Cancer Risk for Patients With Primary Biliary Cholangitis
Source: JAMA Netw Open. 2025 Dec 19;8(12):e2550907. doi: 10.1001/jamanetworkopen.2025.50907 (PMC12717617; doi:10.1001/jamanetworkopen.2025.50907)
Supplement: Supplement 1. — eMethods. [file jamanetwopen-e2550907-s001.pdf]

Supplementary Online Content

Su L, Patel PJ, Shu X. Use of ursodeoxycholic acid and cancer risk for patients with primary biliary cholangitis. *JAMA Netw Open.* 2025;8(12):e2550907. doi:10.1001/jamanetworkopen.2025.50907

eMethods.

This supplementary material has been provided by the authors to give readers additional information about their work.

© 2025 Su L et al. *JAMA Network Open.*

eMethods.

1. Data used in the study

Data for this study were sourced from the TriNetX Research Network (TriNetX LLC, Cambridge, MA) and frozen on July 17, 2025. This Network is a federated, real-time platform that aggregates de-identified electronic health records from participating healthcare organizations worldwide. At the time of analysis, the network included data from 109 healthcare organizations encompassing approximately 156 million unique patients across more than 20 countries spanning the Americas, Europe, Africa, and Asia. The dataset represents diverse populations across geographic regions, age groups, race and ethnicity, income and insurance categories, and clinical care settings. TriNetX, LLC is compliant with the Health Insurance Portability and Accountability Act (HIPAA). Any data displayed on the TriNetX Platform in aggregate form, or any patient-level data provided in a data set generated by the TriNetX Platform only contains de-identified data as per the de-identification standard defined in Section §164.514(a) of the HIPAA Privacy Rule. TriNetX built-in analytic functions (e.g., incidence, prevalence, outcomes analysis, survival analysis, propensity score matching) allow for patient-level analyses, while only reporting population-level data. The University of Maryland, Baltimore Institutional Review Board (IRB) determined research using TriNetX, as described here, does not constitute human subjects research and is therefore exempt from IRB review.

TriNetX maps race and ethnicity data to the following categories: (1) Race: Asian, American Indian or Alaskan Native, Black or African American, Native Hawaiian or Other, White, Unknown race; and (2) Ethnicity: Hispanic or Latino, Not Hispanic or Latino, Unknown Ethnicity.

TriNetX completes an intensive data preprocessing stage to minimize missing values. All covariates are either binary, categorical (which expands to a set of binary columns), or continuous but essentially guaranteed to exist (e.g., age). Missing sex values are coded as "Unknown Sex". The missing data for race and ethnicity are presented as "Unknown race" or "Unknown Ethnicity". For other variables including medical conditions, procedures, lab tests, and socio-economic determinant health, the value is either present or absent so "missing" is not pertinent.

2. Cox Proportional Hazards Model Analysis

The Cox proportional hazards model estimates the effect of exposure on the rate of a specific outcome over time. For each comparison, an index event was defined (i.e., prescription of UDCA or diagnosis of PBC), covariates and an outcome were selected, and time windows relative to the index event were specified. Covariates included for overall GI cancer were age at index, sex, race/ethnicity, family history of primary malignant neoplasm (Z79.1), aspirin use (RXNORM:1191), long-term use of NSAIDs (Z79.1), personal history of other diseases of digestive system (K00-K95), symptoms, signs and abnormal clinical and laboratory findings

© 2025 Su L et al. *JAMA Network Open.*

(R00-R99), endocrine, nutritional and metabolic diseases (E00-E89), potential health hazards related to socioeconomic and psychosocial circumstances (Z55-Z65), personal history of benign neoplasm (Z86.01), problems related to lifestyle (Z72), family history of diabetes mellitus (Z83.3), viral hepatitis B (B16, B18.1 and B19.1), viral hepatitis C (B17.1, B18.2 and B19.2), cirrhosis of liver (K74.60, K74.69 and K70.3), hepatic fibrosis (K74.0), fatty (change of) liver (K76.0), other specified inflammatory liver diseases (K75.8), Crohn's disease (K50), ulcerative colitis (K51), polyp of colon (K63.5), bariatric surgery status (Z98.84), encounter for screening for malignant neoplasms (Z12), benign neoplasm of colon, rectum, anus and anal canal (D12), overweight and obesity (E66), body mass index (Z68.25-29, Z68.3 and Z68.4), gastro-esophageal reflux disease (K21), Barrett's esophagus (K22.7), surgical procedures on the digestive system (CPT:1006964), surgical procedures on the cardiovascular system (CPT:1006056), colonoscopy, flexible (CPT:1022231), cardiovascular medications (VA:CV000), central nervous system medications (VA:CN000), pantoprazole (RXNORM:40790), omeprazole (RXNORM:7646), simethicone (RXNORM:9796), esomeprazole (RXNORM:283742), mesalamine (RXNORM:52582), lansoprazole (RXNORM:17128), and obeticholic acid (RXNORM:1798288). Nearly identical covariates were included for liver and colorectal cancer with a few exceptions due to limited sample sizes. For breast cancer, the adjusted covariates included basic demographics variables, family history of primary malignant neoplasm (Z79.1), aspirin use (RXNORM:1191), long-term use of NSAIDs (Z79.1), overweight and obesity (E66), body mass index (Z68.3), hormone replacement therapy (ICD10CM:Z79.890), screening mammography, bilateral (CPT:77067), diagnostic mammography (CPT:77065 and CPT:77066). The model calculated hazard ratios, confidence intervals, and p-values, assuming proportional hazards and using Efron's method to handle tied events.

3. Code Availability Statement

Cohort study design and associated statistics including propensity-score matching and Kaplan-Meier survival analysis were conducted within the TriNetX Advanced Analytics Platform using built-in functions (R version 4.0.2) with significance set at p-value < 0.05 (two-sided).

© 2025 Su L et al. *JAMA Network Open.*
