# Supplementary material for: Use of Ursodeoxycholic Acid and Cancer Risk for Patients With Primary Biliary Cholangitis
Source: JAMA Netw Open. 2025 Dec 19;8(12):e2550907. doi: 10.1001/jamanetworkopen.2025.50907 (PMC12717617; doi:10.1001/jamanetworkopen.2025.50907)
Supplement: Supplement 2. — Data Sharing Statement [file jamanetwopen-e2550907-s002.pdf]

Data Sharing Statement

Su. Use of Ursodeoxycholic Acid and Cancer Risk for Patients With Primary Biliary Cholangitis. *JAMA Netw Open*. Published December 19, 2025. doi:10.1001/jamanetworkopen.2025.50907

Data
Data available: No

Additional Information
Explanation for why data not available: Access to TriNetX is only granted to researchers and healthcare professionals affiliated with participating institutions.
